# Chilaiditi's Sign Associated with Acute Colonic Pseudo-obstruction: A Radiological Diagnosis

**DOI:** 10.7759/cureus.2351

**Published:** 2018-03-20

**Authors:** Taseen Syed, Samid Farooqui, Rutaba Tajammal, Sultan Mahmood, Donald Kastens

**Affiliations:** 1 Internal Medicine, University of Oklahoma Health Sciences Center; 2 Gastroenterology Fellow Section of Digestive Diseases & Nutrition, University of Oklahoma Health Sciences Center; 3 Associate Professor of Medicine Section of Digestive Diseases & Nutrition, University of Oklahoma Health Sciences Center

**Keywords:** chilaiditi, pseudo-obstruction

## Abstract

Chilaiditi’s sign is a rare radiological anomaly of hepato-diaphragmatic interposition of the bowel. We report a case of Chilaiditi’s sign associated with acute colonic pseudo-obstruction.

A 90-year-old male was admitted for hypertensive emergency. His physical examination showed a distended abdomen, decreased bowel sounds, and right upper quadrant tenderness. A chest radiograph demonstrated marked elevation of the right diaphragm and interposition of the hepatic flexure of the colon between the diaphragm and the liver, along with marked gaseous distension up to 9 cm in the ascending colon without any small bowel distension. The patient was managed conservatively with bowel rest, stool softeners, enemas, and intravenous (IV) hydration. The patient improved clinically with resolution of colonic distension.

Chilaiditi's sign and Chilaiditi syndrome are rare entities and therefore are often misdiagnosed and mismanaged. Awareness of the radiological sign, the syndrome itself, and the association with acute colonic pseudo-obstruction is important for all care providers so that they can opt for more conservative management strategies instead of unnecessary interventions including surgeries.

## Introduction

Chilaiditi syndrome is a rare medical condition characterized by the radiological anomaly of hepato-diaphragmatic interposition of the colon along with clinical symptoms. The radiological appearance, without symptoms, is termed Chilaiditi’s sign. Acute colonic pseudo-obstruction (Ogilvie’s Syndrome) is a rare association that can further complicate the management. The diagnostic significance of this rare syndrome lies in its serious complications including intestinal obstruction, perforation, toxic megacolon, and ischemia. We call attention to the significance of this radiological clinical sign when associated with colonic pseudo-obstruction (Ogilvie’s syndrome).

## Case presentation

**Figure 1:**
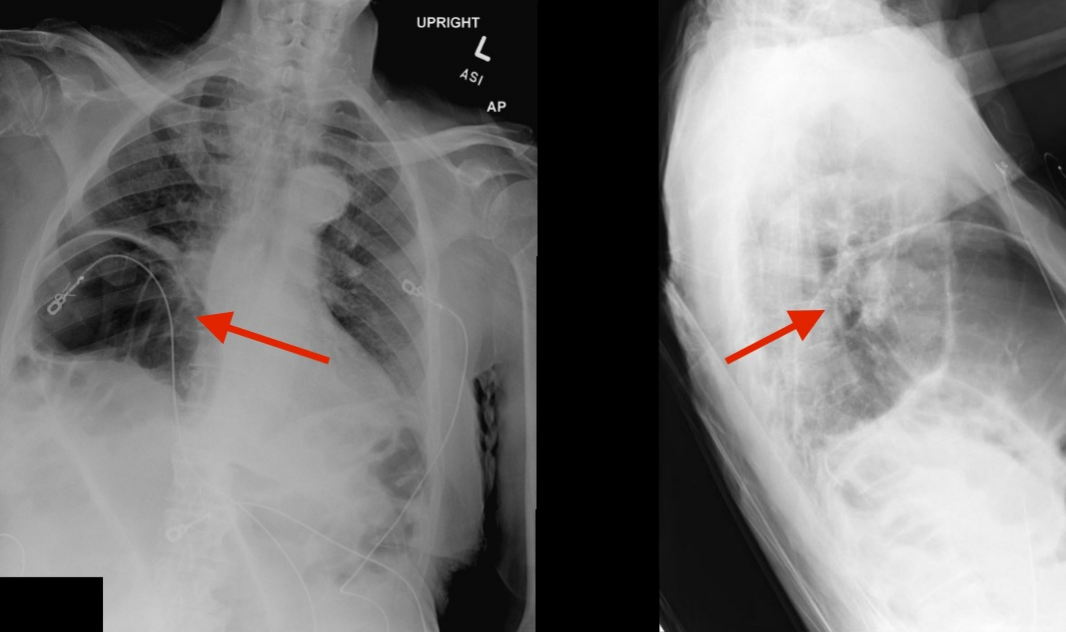
Chest radiograph (anteroposterior and lateral views) showing interposition of the hepatic flexure of the colon between the diaphragm and the liver (Chilaiditi's sign).

**Figure 2:**
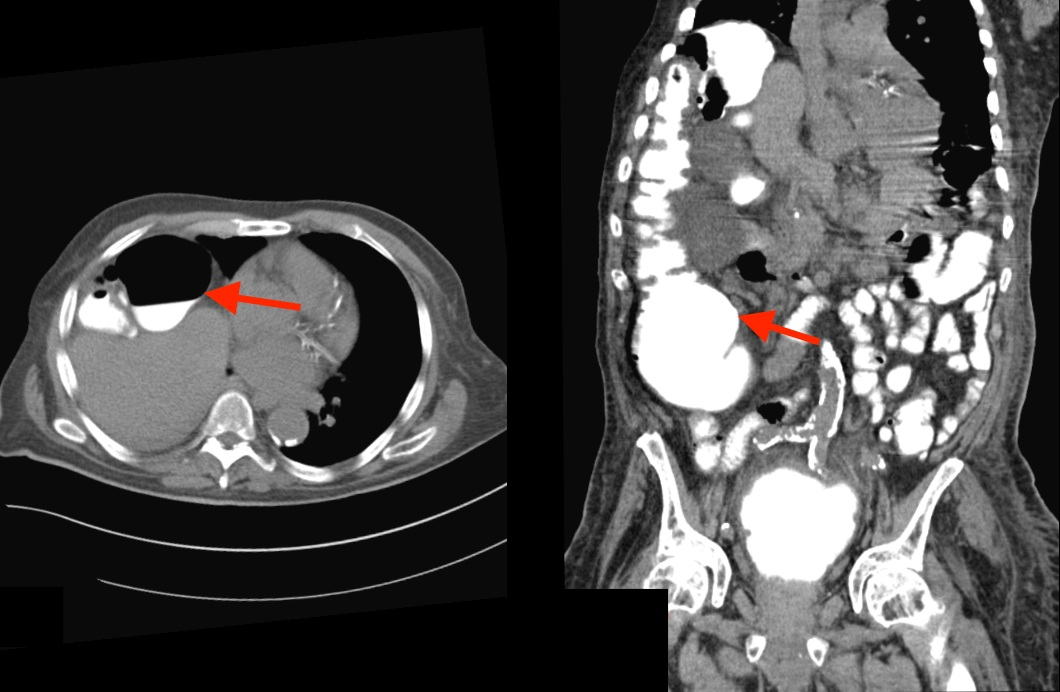
Abdominal computed tomography scan (axial and coronal views) with abdominal contrast showing colonic pseudo-obstruction.

**Figure 3:**
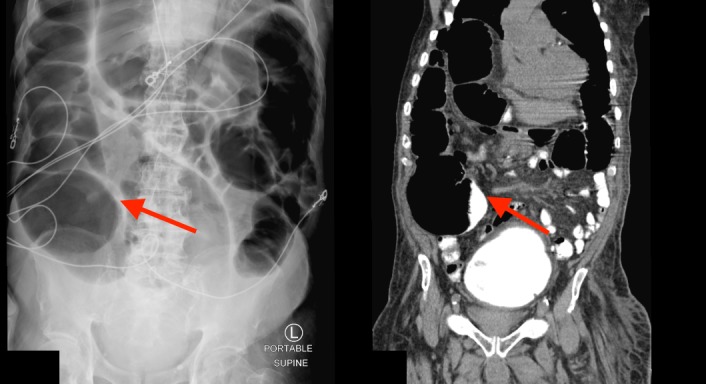
Abdominal radiograph and computed tomography scan (sagittal view) showing colonic pseudo-obstruction.

A 90-year-old male with a history of diabetes mellitus, hypertension, hyperlipidemia, chronic kidney disease stage 2, and stroke was admitted for hypertensive emergency with a blood pressure of 200/105 mmHg, altered sensorium, and acute kidney injury. His physical examination showed bilateral crackles, a distended abdomen, decreased bowel sounds, and right upper quadrant tenderness. Blood workup showed a white cell count of 10.5 K/cmm3, a hemoglobin level of 13 g/dl, a platelet count of 185,000/cmm3, a creatinine level of 1.12 mg/dl, a potassium level of 2.7 mEq/L, a magnesium level of 1.8 mEq/L, and a thyroid stimulating hormone (TSH) level of 1.75 U/mL. On imaging, the chest radiograph demonstrated marked elevation of the right diaphragm and interposition of the hepatic flexure of the colon between the diaphragm and the liver, along with marked gaseous distension of the colon in the upper abdomen (Figure 1). His blood pressure was initially managed with intravenous nicardipine drip that was later transitioned to oral calcium channel blockers, beta blockers, and thiazide diuretics. For his abdominal symptoms, he was treated conservatively with supportive management: kept NPO (nil per os/ nothing through the mouth), intravenous fluids, and electrolyte replacement. All anticholinergic medications and calcium channel blockers were discontinued. The patient did not show any symptomatic improvement with 48 hours of conservative management, at which point an abdominal computed tomography (CT) was obtained with oral contrast, which was consistent with pseudo-obstruction. After reviewing the images, a decision was made to continue close monitoring with no immediate need for surgical decompression (Figures 2, 3). On day 5 of admission, the patient improved clinically with resolution of the abdominal pain, constipation, and bloating. He tolerated oral intake and was discharged home a day later with a follow-up outpatient visit, which he failed to show-up for.

## Discussion

​​Chilaiditi’s sign is a rare radiographic finding named after the Greek radiologist Demetrius Chilaiditi who described it in 1910. The reported incidence of Chilaiditi’s sign (or interpositio hepato-diaphragmatica) on chest and abdominal films is 0.25%–0.28% with a greater male predominance (male to female, 4:1). This sign is reported in 1.18%–2.4% of abdominal computed tomography (CT) scans [1]. In contrast, the Chilaiditi’s syndrome, in addition to the radiographic findings, includes clinical symptoms such as abdominal pain, nausea, vomiting, bloating, constipation, and, less frequently, respiratory distress or chest pain [2]. This radiological sign can be misleading for other disease entities like diaphragmatic hernia, sub diaphragmatic abscess, or bowel perforation (pneumoperitoneum), leading to unnecessary surgeries [3]. In our case, Chilaiditi’s sign was associated with Ogilivie’s syndrome, only requiring conservative management.

In Chilaiditi’s syndrome, the hepatic flexure is the most common portion of the colon that gets impinged between the diaphragm and the liver and the second most common being the ascending or transverse colon. There are some reported cases of small intestine involvement as well [4]. The etiology can be either congenital or acquired. Congenital cases are due to variations in normal anatomy causing interposition of the colon. Acquired cases can be due to paralysis of the diaphragm due to nerve injury, small liver due to cirrhosis, ascites, obesity, chronic constipation causing anatomic distortion, or diaphragmatic muscular degeneration. It is reported more commonly in the elderly population, in approximately 1% of adults [1, 5, 6]. Complications may include volvulus, perforation, toxic megacolon, intestinal obstruction, pseudo-obstruction, and perforated subdiaphragmatic appendicitis [6].

Acute colonic pseudo-obstruction, also known as Ogilvie's syndrome (named after Sir William Ogilvie, who described this phenomenon initially in 1948), refers to dilation of the colon without the presence of any mechanical obstruction [7]. Old age, multiple comorbidities, major surgical procedure, and presence of an acute illness are the common factors associated with the development of Ogilvie’s syndrome. Recurrence has been reported in 17-38% of cases after initial success with neostigmine [8]. Our patient was an elderly male with multiple comorbidities including hypertensive emergency and we believe that all these factors contributed in the development of Ogilvie’s syndrome, which manifested as Chilaiditi’s sign on radiological imaging.

No treatment is required for an asymptomatic patient with Chilaiditi's sign as this is an incidental finding. Chilaiditi syndrome, if present, is usually treated conservatively with bowel rest, nasogastric decompression, stool softeners, enemas, and IV hydration, with surgical intervention reserved only when there is concern for volvulus, perforation, or ischemia. A repeat radiograph may confirm successful decompression of the bowel with no air below the hemidiaphragm. When Chilaiditi's sign presents with Ogilvie's syndrome, it is managed with correcting the predisposing factors worsening colonic pseudo-obstruction such as electrolyte disturbances and anticholinergic medications. Treatment options include administration of neostigmine or radiological decompression [9]. Surgical interventions include bowel resection, colopexy, and/or hepatopexy [3].

## Conclusions

Chilaiditi's sign and Chilaiditi syndrome are rare entities and therefore are often misdiagnosed and mismanaged. Awareness of the radiological sign, the syndrome itself, and the association with Oglivie's syndrome is important for all care providers so that they opt for more conservative management strategies instead of unnecessary interventions including surgeries.
